# Cross-Reactive Antibodies to Pandemic (H1N1) 2009 Virus, Singapore

**DOI:** 10.3201/eid1605.091678

**Published:** 2010-05

**Authors:** Julian W. Tang, Paul A. Tambyah, Annelies Wilder-Smith, Kim-Yoong Puong, Robert Shaw, Ian G. Barr, Kwai-Peng Chan

**Affiliations:** National University Hospital, Singapore (J.W. Tang); National University of Singapore, Singapore (P.A. Tambyah, A. Wilder-Smith); Singapore General Hospital, Singapore (K.-Y. Puong, K.-P. Chan); World Health Organization Laboratory for Influenza Reference and Research, Melbourne, Victoria, Australia (R. Shaw, I. Barr)

**Keywords:** Influenza, cross-reactive antibodies, seroprevalence, pandemic (H1N1) 2009, vaccine, swine, Singapore, viruses, letter

**To the Editor:** Accumulating evidence suggests that the degree of serologic cross-reactivity to pandemic (H1N1) 2009 virus varies between populations worldwide. To assess potential serologic cross-reactivity in Singapore, we obtained serum samples during May–June 2009 from 50 randomly recruited, healthy volunteers born mostly before 1958 (i.e., potentially those with some natural exposure to the then circulating H1N1/1918-like subtype viruses) before widespread transmission of pandemic (H1N1) 2009 virus in Singapore. Standard serologic hemagglutination-inhibition (HI) tests (*1*) were performed in 2 reference laboratories (Singapore during July–October 2009 and Melbourne, Australia, in January 2010), and microneutralization (MN) tests (*2*) were performed in 1 reference laboratory (Singapore) for each serum sample against pandemic (H1N1) 2009 virus (A/Auckland/1/2009) and seasonal influenza (H1N1) virus (A/Brisbane/59/2007). The study was reviewed and approved by the National Healthcare Group Domain-Specific Review Board (ref no. E/09/289, J.W.T. principal investigator).

Mean ± SD age of participants was 60.1 ± 7.4 years (range 45–82 years); 31 (62%) were women, 42 (84%) were born in Singapore (the rest in Hong Kong, Malaysia, or India), and 26 (52%) had not traveled outside Singapore. None of the 50 participants had HI or MN titers >40 against influenza A/Auckland/1/2009 when samples were tested in either laboratory. In contrast, 18 samples had either HI or MN titers >40 against seasonal influenza A/Brisbane/59/2007 (Table). Use of guinea pig or turkey erythrocytes in HI assays had little effect on the results (Table). Thus, our results are similar to those of Chen et al. (*3*) and Itoh et al. (*4*) for this small cohort in that none of the participants 40–80 years of age from Southeast Asia had cross-reactive antibodies to pandemic (H1N1) 2009 virus.

**Table:** Cross-reactive antibody titers to pandemic (H1N1) 2009 and seasonal influenza viruses for 50 persons, Singapore, May–October 2009*

Patient age, y/sex	Laboratory 1 (Singapore General Hospital)						Laboratory 2 (WHO, Melbourne)^+^			
	A/Auckland/1/2009			A/Brisbane/59/2007			A/Auckland/1/2009			A/Brisbane/59/2007
	HI†	MN		HI†	MN		HI‡	HI†		HI‡
45/F	<10	<10		<10	<10		<10	<10		<10
52/F	<10	10		10	10		<10	10		<10
52/F	<10	<10		10	<10		<10	<10		<10
52/F	<10	<10		10	<10		<10	<10		<10
53/M	<10	<10		10	<10		<10	<10		<10
53/F	<10	<10		20	40		<10	<10		40
53/F	<10	<10		160	80		<10	<10		160
53/M	<10	<10		10	<10		<10	<10		<10
53/F	<10	<10		160	320		<10	<10		160
53/F	<10	<10		10	<10		<10	<10		<10
54/F	<10	<10		10	<10		<10	<10		<10
54/M	10	<10		10	<10		<10	20		<10
55/F	<10	<10		10	<10		<10	10		<10
55/F	<10	<10		160	40		<10	<10		160
55/M	<10	<10		10	<10		<10	<10		<10
56/F	<10	<10		20	10		<10	<10		10
56/F	<10	<10		80	80		<10	20		40
56/F	<10	10		10	40		<10	<10		10
56/F	<10	<10		10	<10		<10	<10		<10
57/M	10	20		40	40		20	20		<10
58/M	<10	<10		20	20		<10	10		20
58/F	<10	<10		40	20		<10	<10		80
59/M	<10	<10		10	<10		<10	<10		<10
59/M	<10	<10		20	20		20	20		10
59/F	<10	<10		40	40		<10	<10		40
60/M	<10	<10		80	40		<10	<10		80
60/M	<10	<10		20	10		<10	<10		20
60/F	10	<10		10	<10		NR	<10		<10
61/F	10	<10		10	<10		<10	10		<10
61/M	10	<10		160	160		<10	<10		320
61/M	<10	<10		<10	<10		<10	<10		<10
61/F	<10	<10		10	<10		<10	<10		<10
62/F	<10	20		10	20		NR	NR		NR
62/F	10	10		80	20		<10	<10		40
62/F	<10	<10		20	10		<10	20		40
62/F	<10	<10		80	40		<10	<10		80
63/M	<10	<10		80	40		<10	<10		80
63/F	<10	<10		10	<10		<10	<10		<10
63/M	<10	<10		40	20		<10	<10		20
63/M	<10	<10		40	80		<10	<10		20
64/F	10	<10		20	10		<10	10		10
65/F	10	<10		10	<10		<10	20		<10
66/M	<10	<10		<10	<10		<10	<10		<10
68/M	<10	<10		40	80		<10	<10		40
71/F	10	10		10	20		<10	<10		<10
72/F	<10	20		10	20		10	10		<10
75/M	<10	<10		10	10		<10	20		<10
76/M	<10	<10		40	20		<10	<10		40
78/F	<10	10		10	20		<10	<10		<10
82/F	10	10		20	10		<10	20		<10

*WHO, World Health Organization; HI, hemagglutination inhibition; MN, microneutralization; NR, no results (because of insufficient serum). †Tested with guinea pig erythrocytes. ‡Tested with turkey erythrocytes.

Although differences in population demographics and laboratory methods used make comparisons between studies difficult, one of the most striking observations from various studies has been the higher levels of cross-reactive antibody titers in prepandemic serum samples from older persons (>80 years of age) in western populations (United States and United Kingdom) (*5*,*6*) than from persons in eastern populations (China) (*3*) and Singapore (this study). Although Itoh et al (*4*) did not find serologic cross-reactivity in the population <80 years of age in Japan, they found higher levels of cross-reactive antibodies in their population >80 years of age. Historically, because epidemiologic data suggest that influenza (H1N1)/1918–like viruses were widespread in Asia, these contrasting results are a stimulus for additional large-scale studies to assess the effect of these viruses in these populations.

Although the main limitation of our study is the small sample size, several reasons may account for different findings in population studies of serologic cross-reactivity. First, populations may not be comparable in terms of geographic proximity and their potential for community-acquired infection within the same wave of a seasonal influenza epidemic with a virus that was similar to pandemic (H1N1) 2009 virus. Chen et al. (*3*) reported that their serum samples were obtained mainly from rural farmers in China who lived farther apart than city dwellers, However, Hancock et al. (*5*) reported that their samples were obtained from vaccine trials conducted in 1976 or 2005–2009 involving academic, government, and industrial workers, which likely indicates that these persons were urban-based (i.e., living and working more closely to each other than rural farmers in China). Thus, results of our study may not be directly comparable with either of these previous studies because our population resided in Southeast Asia and was urban-based.

Second, use of seasonal influenza vaccine has varied in different populations, with Singapore having one of the lowest recorded use rates in the Western Pacific region, and far lower than that in the United States (*6*). If previous seasonal influenza viruses shared a degree of antigenic cross-reactivity with pandemic (H1N1) 2009 virus, contemporary seasonal influenza vaccines, if well-matched, should reflect changing antigenicity of seasonal influenza viruses; thus, vaccinated populations may have acquired some serologic cross-reactivity through previous influenza vaccines. However, it is likely that past infection rather than vaccination results in cross-reactivity, as suggested by Miller et al. (*7*).

Third, because pandemic (H1N1) 2009 virus originated mainly from swine viruses in North America and Europe (*8*), resident populations in these countries have been exposed to these virus lineages more frequently than populations in Asia, and therefore may have acquired a greater degree of preexisting cross-reactive immunity to pandemic (H1N1) 2009 virus. A recent review of human swine influenza infections suggests that they may not be uncommon (*9*), although the true incidence of human infections with swine influenza is unknown because of paucity of swine influenza surveillance data worldwide (*8*).

In conclusion, partial cross-immunity and cell-mediated immunity may be present but not detected by HI or MN assays. Thus, results of standard serologic assays may not be providing all relevant data (*10*).
